# Atrial fibrillation fingerprinting; spotting bio‐electrical markers to early recognize atrial fibrillation by the use of a bottom‐up approach (AFFIP): Rationale and design

**DOI:** 10.1002/clc.23370

**Published:** 2020-04-18

**Authors:** Roeliene Starreveld, Paul Knops, Kennedy S. Ramos, Maarten C. Roos‐Serote, Ad J.J.C. Bogers, Bianca J.J.M. Brundel, Natasja M.S. de Groot

**Affiliations:** ^1^ Department of Cardiology Erasmus Medical Center Rotterdam The Netherlands; ^2^ Department of Cardiothoracic Surgery Erasmus Medical Center Rotterdam The Netherlands; ^3^ Department of Physiology, Amsterdam UMC Vrije Universiteit Amsterdam, Amsterdam Cardiovascular Sciences Amsterdam UK

**Keywords:** atrial fibrillation, biomarkers, cardiac electrophysiology, electrogram fractionation, high density mapping, signal modeling

## Abstract

**Background:**

The exact pathophysiology of atrial fibrillation (AF) remains incompletely understood and treatment of AF is associated with high recurrence rates. Persistence of AF is rooted in the presence of electropathology, defined as complex electrical conduction disorders caused by structural damage of atrial tissue. The atrial fibrillation fingerprinting (AFFIP) study aims to characterize electropathology, enabling development of a novel diagnostic instrument to predict AF onset and early progression.

**Hypotheses:**

History of AF, development of post‐operative AF, age, gender, underlying heart disease, and other clinical characteristics impact the degree of electropathology.

**Methods:**

This study is a prospective observational study with a planned duration of 48 months. Three study groups are defined: (1) patients with (longstanding) persistent AF, (2) patients with paroxysmal AF, and (3) patients without a history of AF, all undergoing open‐chest cardiac surgery. Intra‐operative high‐resolution epicardial mapping is performed to identify the patient‐specific electrical profile, whereas the patient‐specific biological profile is assessed by evaluating proteostasis markers in blood samples and atrial appendage tissue samples. Post‐operative continuous rhythm monitoring is performed for detection of early post‐operative AF. Late post‐operative AF (during 5‐year follow‐up) is documented by either electrocardiogram or 24‐hour Holter registration.

**Results:**

The required sample size for this study is estimated at 447 patients. Up till now, 105 patients were included, of whom 36 have a history of AF.

**Conclusion:**

The AFFIP study will elucidate whether electrophysiological and structural characteristics represent a novel diagnostic tool, the AF fingerprint, to predict onset and early progression of AF in cardiac surgery patients.

## 1 INTRODUCTION

Atrial fibrillation (AF) is the most common cardiac arrhythmia worldwide, and its incidence^1^ and associated medical health care costs^2^ continuously increase. Even though numerous predisposing factors for progression of AF have been identified, the exact pathophysiology remains incompletely understood and treatment of AF is associated with high recurrence rates. As disease progression from recurrent intermittent episodes to finally permanent AF is accompanied by a gradual increase in therapy failure, early recognition of AF is of prime importance. Persistence of AF is rooted in the presence of electropathology, which is defined as complex electrical conduction disorders caused by structural damage of atrial tissue. Therefore, early recognition of AF susceptibility in patients is necessary to halt electropathology and hence disease onset and progression. Although promising artificial intelligence applications are emerging,^3^ up till this day in clinical practice, AF is diagnosed with a surface electrocardiogram when a patient already suffers from AF. This rhythm registration cannot assess the degree of electropathology and thus the stage of AF which is essential for selection of the appropriate therapy. Hence, early recognition of AF and the start of effective treatment is seriously hampered. By characterizing electropathology, we aim to develop a novel diagnostic instrument to predict AF onset and early progression. We hypothesize that every patient has a unique biological and electrical signal profile that is influenced by age, gender and underlying heart disease. This bio‐electrical profile is deduced from the ratio abnormal/normal electrical signals in the atria by utilizing a unique high‐density atrial mapping approach and determination of proteostasis markers in tissue or blood samples related to structural damage. These outcomes are summarized in an AF Fingerprint.

Multi‐site high density epicardial mapping has been used in multiple research protocols in Rotterdam (QUASAR study MEC 2010‐054, HALT&REVERSE study MEC 2015‐393), Leiden and Maastricht.^4^, ^5^, ^6^, ^7^ Since 2010, the mapping procedure is daily practice in the Erasmus Medical Center. Atrial conduction during both sinus rhythm (SR) and (induced) AF can be visualized to identify the patient‐specific electrical profile. This alone however does not clarify electropathological changes on structural level that contribute to substrate for AF. Previous studies revealed that structural damage is caused by derailment of protein homeostasis due to loss of key modulators within the protein quality system.^8^ Failure of protein quality control in AF involves impairment of heat shock proteins (HSPs),^9^ autophagy,^10^ loss of sarcomeric and microtubule proteins,^11^, ^12^ and activation of DNA damage/PARP1/NAD axis,^13^ favoring progression of AF.

**FIGURE 1:**
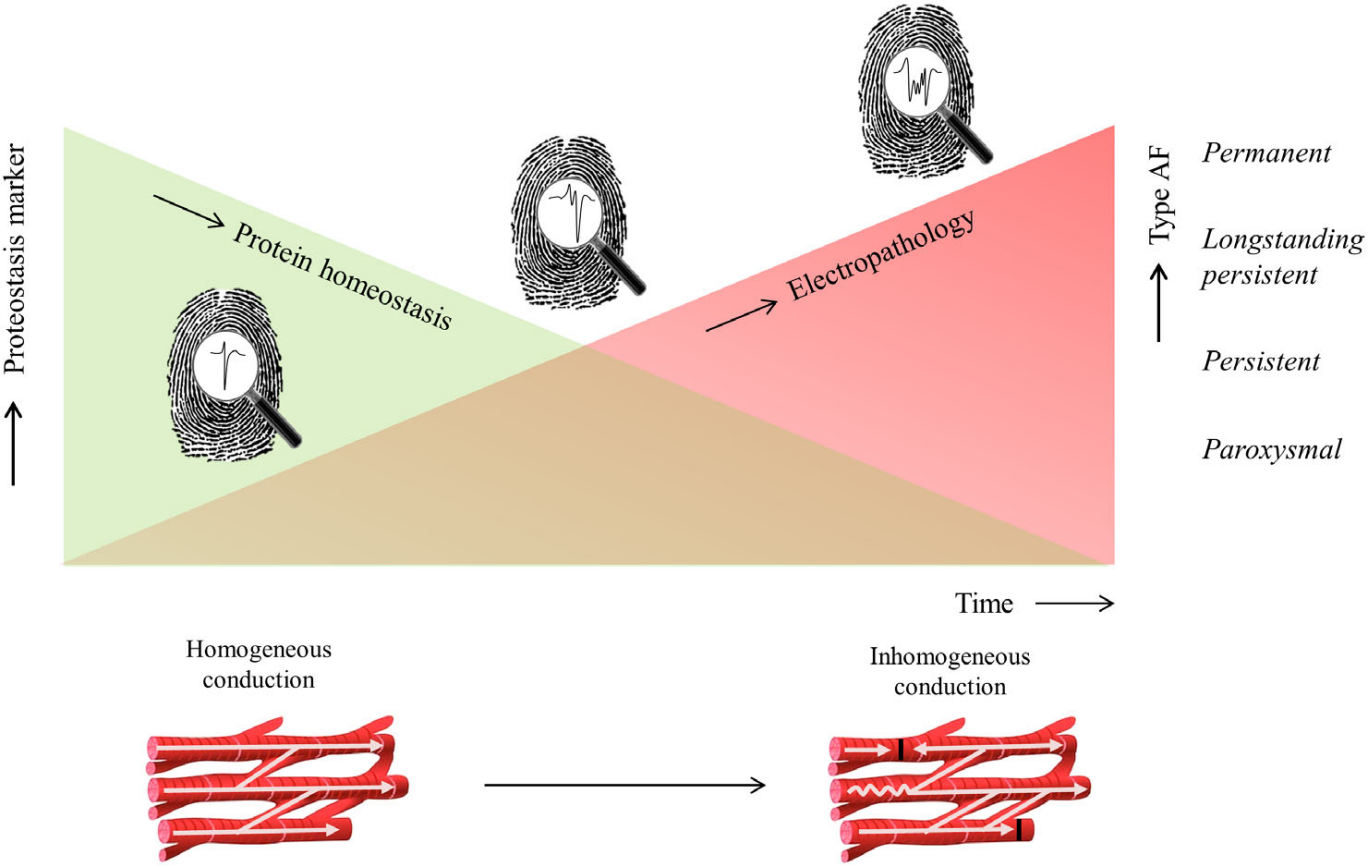
Concept of the atrial fibrillation fingerprinting (AFFIP) study. The AFFIP study hypothesizes that every patient has a unique biological and electrical signal profile that is influenced by age, gender and heart disease. Intra‐operative high‐resolution epicardial mapping is performed to identify the patient‐specific electrical profile, whereas the biological profile is assessed by evaluating proteostasis levels in blood samples and atrial appendage tissue samples. Derailment of protein homeostasis can lead to structural remodeling, favoring inhomogeneous conduction and progression of atrial fibrillation (AF). We aim to develop a novel diagnostic tool, the bio‐electrical AF Fingerprint, to predict onset and early progression of AF

The AFFIP study combines electrophysiological and structural alterations into one AF fingerprint (Figure 1). Electrophysiological data obtained from epicardial mapping during surgery are combined with proteostasis markers on one hand and atrial tissue characteristics on the other hand. By comparing the bio‐electrical AF fingerprint of patients from different age groups, gender, history of AF, development of post‐operative AF, underlying heart disease and other clinical characteristics, we hope to gain more insight in the mechanism underlying AF and the development of a substrate for AF. The findings will elucidate whether structural and electrophysiological characteristics represent a novel diagnostic tool, the AF fingerprint, to predict onset and early progression of AF in cardiac surgery patients.

## 2 METHODS

AFFIP is a prospective observational study, with a planned duration of 48 months. This study is carried out according to the principals of the Declaration of Helsinki and in accordance with the Medical Research involving Human Subjects Acts. The study is part of the HALT&REVERSE protocol which is approved by the Rotterdam local medical ethical committee (MEC‐2014‐393).

### 2.1 Study objectives

The primary study objectives are to test the correlation between the AF fingerprint, revealing the degree of electropathology including for example, patterns of activation and signal morphology, proteostasis levels and atrial tissue characteristics, clinical characteristics and onset and progression of AF in patients undergoing open‐chest cardiac surgery.

### 2.2 Study population

Patients with structural heart disease scheduled for elective cardiac surgery are included. The study population consists of three study groups: patients with (longstanding) persistent AF (group 1), patients with paroxysmal AF (group 2) and patients without a history of AF (group 3) undergoing open‐chest cardiac surgery. In line with ESC guidelines, patients with documentation (ECG or ECG description) of self‐terminating AF episodes up to 7 days, or with AF episodes cardioverted within 7 days are classified as paroxysmal AF. Patients with documentation of AF episodes longer than 7 days or longer than a year are classified as persistent and longstanding persistent AF, respectively. Patients are recruited at the Department of Cardiothoracic Surgery at the Erasmus Medical Center, Rotterdam, The Netherlands. Prior to enrolling in the study, each patient is provided an oral and a written explanation of the study procedure. Written informed consent is obtained from all patients.

**FIGURE 2:**
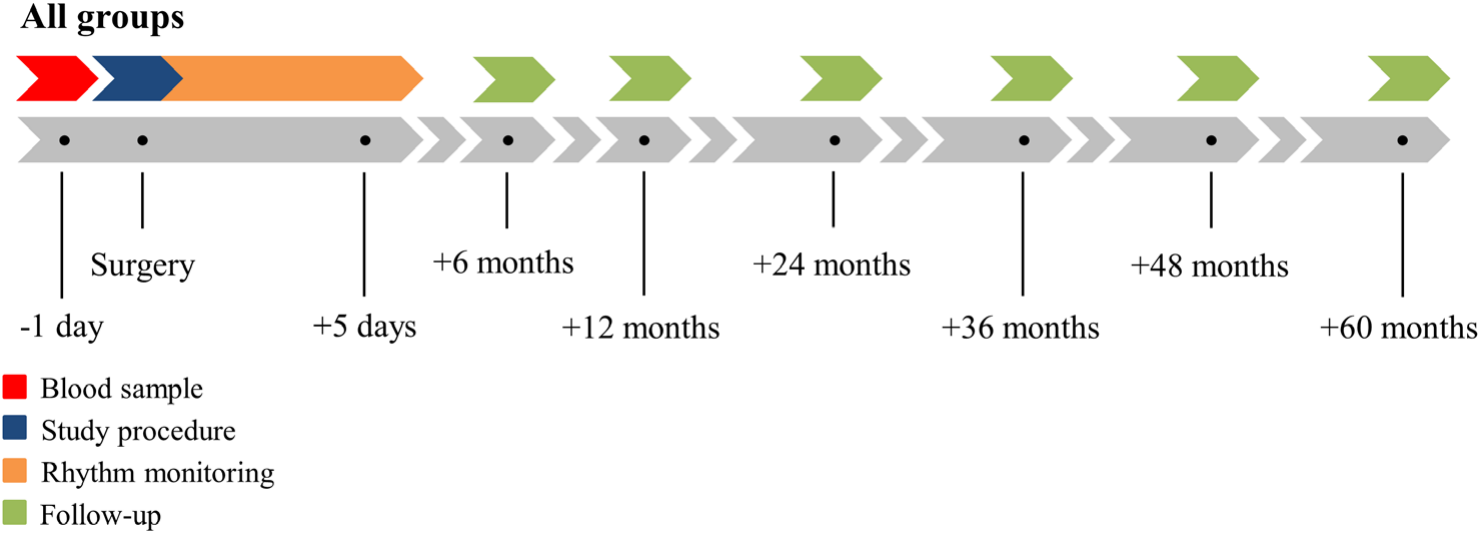
Time course of the atrial fibrillation fingerprinting (AFFIP) study. A baseline blood sample (red bar) is obtained from all patients 1 day prior to surgery. During surgery, the study procedure (blue bar, epicardial mapping) is performed, followed by post‐operative continuous rhythm monitoring (orange bar). Patients are consulted by phone at 6 months, 12 months, and yearly up to 5 years after surgery for detection of late post‐operative atrial fibrillation (AF) (green bars)

Prior to cardiac surgery, blood samples are taken from all patients for determination of proteostasis levels (Figure 2). Patient characteristics (eg, age, medical history, and cardiovascular risk factors) are obtained from the patient's file.

### 2.3 Inclusion criteria

In order to be eligible to participate in this study, a subject must meet all of the following criteria:

- at least 18 years of age;
- structural heart disease (with or without history of AF); and
- scheduled for elective cardiac surgery.

### 2.4 Exclusion criteria

A potential subject who meets any of the following criteria is excluded from participation in this study:

- hemodynamic instability;
- emergency cardiac surgery; and
- redo‐cardiac surgery.

Additional details of entry criteria are listed in the Appendix S1.

### 2.5 Intra‐operative mapping procedure

**FIGURE 3:**
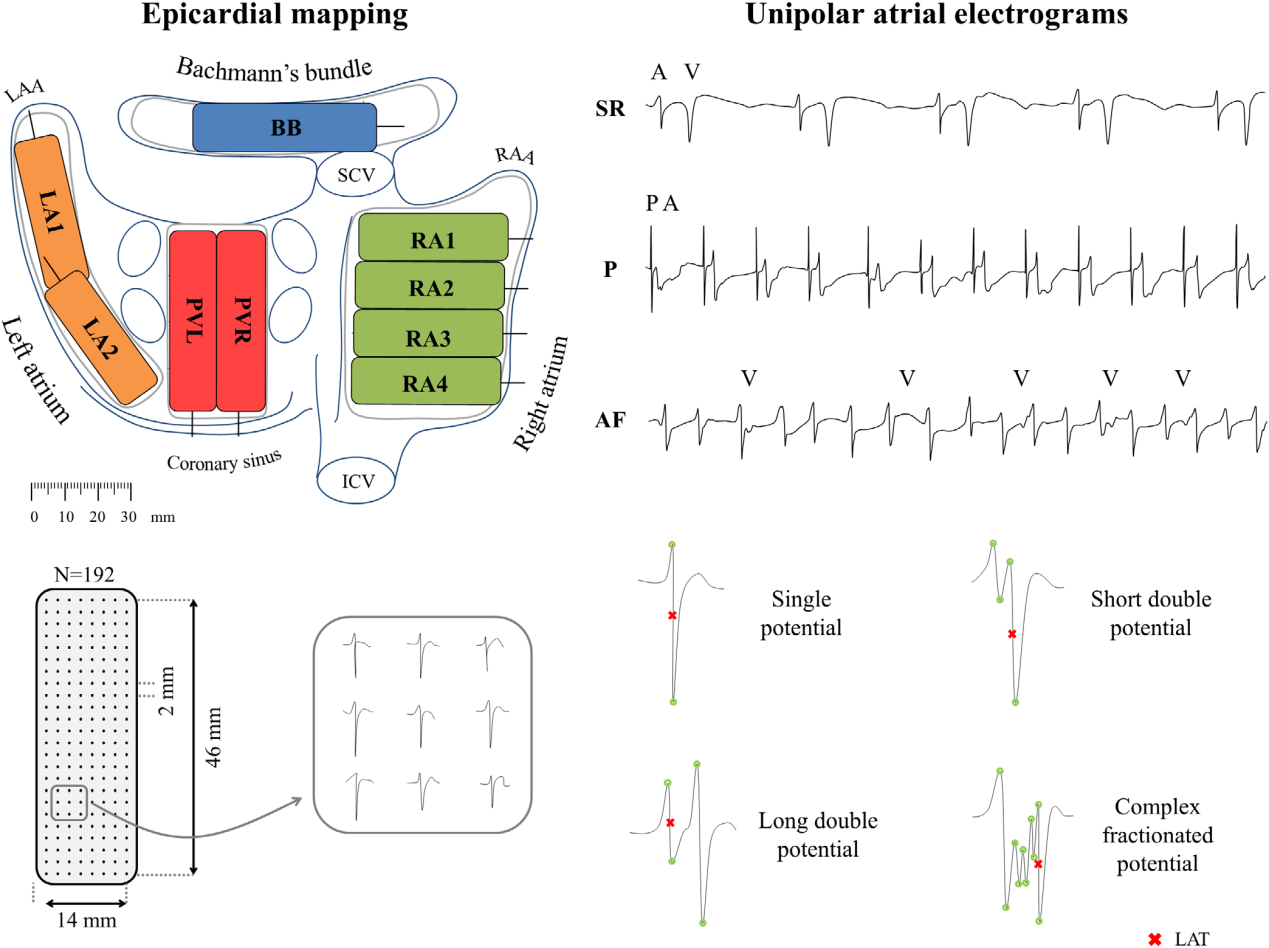
Epicardial mapping to retrieve unipolar atrial electrograms. With the use of a 192 electrode arrays, the left atrium (LA), right atrium (RA), pulmonary vein (PV) area, and Bachmann's bundle (BB) are mapped following this predefined mapping scheme. Unipolar atrial electrograms are collected during sinus rhythm (SR), pacing maneuvers (P), and (electrically induced) atrial fibrillation (AF). Morphology of atrial potentials are subdivided into four categories: single potentials (one deflection), short double potentials (two deflections less than 15 ms apart), long double potentials (two deflections 15 ms or more apart), and complex fractionated potentials (three or more deflections). ICV, inferior caval vein; LAA, left atrial appendage; PVL, left pulmonary vein area; PVR, right pulmonary vein area; RAA, right atrial appendage; SCV, superior caval vein

Epicardial mapping is performed during surgery. Patients are under full anesthesia and vital signs are monitored continuously throughout the procedure. Epicardial unipolar electrograms are recorded using a custom‐made multi‐site electrode array. Recordings are made at nine consecutive sites (right atrium 1‐4, Bachmann's bundle, right and left pulmonary vein, and left atrium 1‐2), following a predefined mapping scheme (Figure 3) during SR (nine sites, 5 seconds/site), during pacing maneuvers for inducing AF (one site, Bachmann's bundle), and in AF (nine sites, 10 seconds/site). Pacing is performed with atrial fixed rate pacing directly from the electrode or with a standard temporary pacemaker wire. If AF sustains at the end of the mapping procedure, SR is restored with 5‐10 J electrical cardioversion.

After introduction of the extra corporal circulation into the right atrium via the right atrial appendage (RAA) a tissue sample (approximately 10 × 10mm) is obtained from the incision site in all patients. In patients undergoing mitral valve surgery, the left atrial appendage (LAA) is also incised and a small tissue sample is excised. In patients undergoing surgical pulmonary vein isolation, a left sided procedure in patients with AF which includes amputation of the LAA, the LAA tissue is also studied.

### 2.6 Follow‐up

After procedure, the heart rhythm is continuously monitored until hospital discharge in order to detect early post‐operative AF. Patients are also consulted by phone at 6 months, 12 months, and yearly up to 5 years after surgery in order to detect late post‐operative AF (Figure 2). If post‐operative AF is suspected, documentation of electrocardiography, 24‐hour Holter registration or a clinical discharge letter from peripheral hospitals are retrieved.

### 2.7 Tissue analysis

All obtained blood samples and atrial tissue samples are stored at ‐80°C until transport to the Amsterdam UMC, location VU Medical Center. Proteostasis markers include HSP27, HSP70, HSPA1A, HSPA5, HSPB1, HSPB5, HSPB6, HSPB7, HSPB8, HSPD1, α‐SMA, LC3B‐II, TIMP1, LOX3, MMP9, Galectin‐3, NCAM, MT‐ND1, and COX3, and are determined by commercially available ELISAs and Western blot analysis at the Department of Physiology of the Amsterdam UMC.

### 2.8 Main study parameters and endpoints

Primary endpoint of the study is development or recurrence of AF. Secondary endpoints include implantation of an atrial pacemaker or implantable cardioverter defibrillator. Additional secondary endpoints are described in the Appendix S1. At present, no substudies are planned.

**FIGURE 4:**
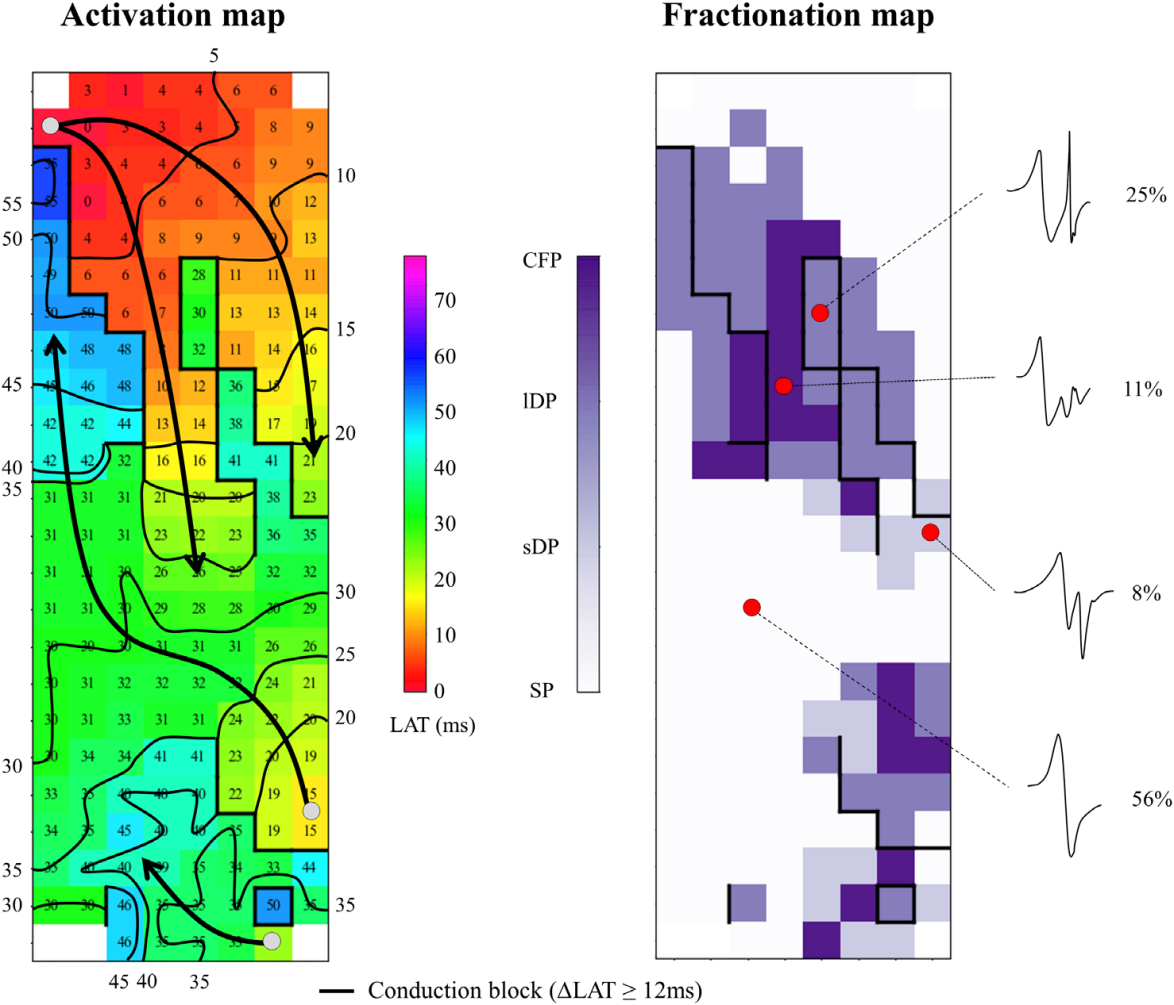
Construction of the atrial fibrillation fingerprint (AFFIP). The left panel shows an example of an activation map during electrically induced atrial fibrillation (AF) of the left pulmonary vein area in a patient undergoing coronary artery bypass grafting without a history of AF. Isochrones are drawn at 5 ms, areas of conduction block (Δ local activation time [LAT] ≥ 12 ms) are indicated by black bars and the origin of peripheral waves by gray dots. The arrows indicate main activation direction. The corresponding fractionation map is displayed in the right panel and shows the different types of atrial potential morphologies: single potentials (SP, one deflection), short double potentials (sDP, two deflections less than 15 ms apart), long double potentials (lDP, two deflections 15 ms or more apart) and complex fractionated potentials (CFP, three or more deflections). The maps are used to determine the incidence of for example, fractionation and conduction block

Deflections of the recorded atrial electrograms are detected semi‐automatically in custom‐made Python 3.6 software. In case of fractionated electrograms, the component with the steepest negative slope is taken as the local activation time (Figure 3). Electrograms with injury potentials and artifacts are excluded from analysis by consensus of two investigators. Signals are used to construct color‐coded activation, conduction block, break‐through wave, fractionation, and voltage maps (Figure 4). Furthermore, the relation between patterns of activation, incidence of breakthrough waves, fractionation, fibrillation intervals, conduction abnormalities, and voltage is studied and compared between the different atrial sites and atrial rhythms. Electrical changes as defined with the above‐mentioned electrical parameters will be correlated with the pre‐operative proteostasis levels, development of post‐operative AF, age, gender, and other clinical characteristics.

The primary hypothesis is that the degree of electropathology will be increased in patients with a history of AF and patients whom develop post‐operative AF. Secondarily, we hypothesize that age, gender, underlying heart disease and other clinical characteristics impact the degree of electropathology.

### 2.9 Sample size calculation

Based upon our experience in prior mapping studies approximately 30% of patients develop early post‐operative AF in the Erasmus MC,^13^ which is in correspondence with published literature.^14^

There is no data on the proposed novel electrophysiological parameters and biomarkers, and calculation of the sample size is therefore at present not possible. However, we used data obtained from a pilot study containing five patients with and five patients without AF. With a *P* value of 0.05 and a chosen power of 0.95 the required number is 135 per study group. An attrition rate of 10% increases this to 149 patients in each group. The required sample size for this study is therefore estimated at 447 patients. These calculations will be repeated after the first 50 patients in every group in order to adjust the sample size.

### 2.10 Statistical analysis

Associations between proteostasis markers, electrical signals and clinical patient outcomes are calculated using multivariate logistic regression and cox regression models. Log rank tests compare patient groups with different stages of AF. Continuous and categorical electrophysiological parameters are compared with respectively ANOVA and chi‐square tests. For repeated biomarker measurements, joint modeling and mixed modeling analysis is used. ANOVA with Bonferroni adjustments corrects for analysis of multiple biomarkers.

### 2.11 Study organization

This multi‐disciplinary study is carried out by dedicated teams, whom are responsible for the following tasks:

Translational Electrophysiology Research Unit of the Department of Cardiology at the Erasmus Medical Center, Rotterdam, The Netherlands: patient screening and recruitment, collection of electrophysiological data during intra‐operative mapping procedure, collection of blood samples, patient follow‐up, electrophysiological data analyses, and statistical analyses.

Department of Cardiothoracic Surgery at the Erasmus Medical Center, Rotterdam, The Netherlands: intra‐operative mapping procedure and collection of atrial tissue samples during open‐chest cardiac surgery.

Atrial Fibrillation Research Unit of the Department of Physiology at the Amsterdam UMC, Amsterdam, The Netherlands: analysis of biological markers from tissue and blood samples.

Trial Office of the Department of Cardiothoracic Surgery, Erasmus Medical Center, Rotterdam, The Netherlands: data safety monitoring.

Supervision and steering of these teams is done by N.M.S.d.G., B.J.J.M.B., and A.J.J.C.B. Prof. Eric Boersma of the Department of Clinical Epidemiology at the Erasmus Medical Center, Rotterdam, The Netherlands, supervises all statistical analyses.

A detailed list of team members is included in the Appendix S1.

## 3 RESULTS

The AFFIP study started in January 2017. The first patient enrolled on January 27, 2017, and up till now 105 patients were included (as of March 20, 2020), of whom 36 have a history of AF. Table 1 provides the preliminary baseline characteristics of all enrolled patients.

**TABLE 1:** Preliminary baseline characteristics of enrolled patients (as of March 20, 2020)

Number of patients	105
Male	70 (67%)
Age (years)	64 (54‐71)
BMI	27.2 (24.6‐30.1)
Underlying heart disease	
CABG	16 (15)
AVD	18 (16)
MVD	11 (10)
CABG + AVD	8 (8)
CABG + MVD	4 (4)
AVD + MVD	4 (4)
CHD	44 (42)
History of AF	36 (34)
Paroxysmal	20 (56)
Persistent	14 (39)
Longstanding persistent	2 (6)
Hypertension	48 (46)
Dyslipidemia	26 (25)
Diabetes mellitus	16 (15)

*Note:* Values are presented as N (%) or median (25th to 75th percentile).

Abbreviations: AVD, aortic valve disease; BMI, body mass index; CABG, coronary artery bypass grafting; CHD, congenital heart disease; MVD, mitral valve disease.

## 4 DISCUSSION

In clinical practice, AF is currently diagnosed with a surface electrocardiogram when a patient already suffers from AF. This rhythm registration cannot assess the degree of electropathology and thus the stage of AF which is essential for selection of the appropriate therapy. Hence, early recognition of AF and the start of effective treatment is seriously hampered. The AFFIP study aims to characterize electropathology, enabling development of a novel diagnostic instrument to predict AF onset and early progression. Electrophysiological data obtained from epicardial mapping during surgery are combined with proteostasis markers on one hand and atrial tissue characteristics on the other hand. The findings will elucidate whether electrophysiological and structural characteristics represent a novel diagnostic tool, the AF fingerprint, to predict onset and early progression of AF in patients who had cardiac surgery.

## CONFLICT OF INTEREST

The authors declare that they have no conflicts of interests to disclose.

## Supporting information


**Appendix** **S1:** Supplementary Information
