# Supplementary material for: Atrial fibrillation fingerprinting; spotting bio‐electrical markers to early recognize atrial fibrillation by the use of a bottom‐up approach (AFFIP): Rationale and design
Source: Clin Cardiol. 2020 Apr 18;43(6):546–52. doi: 10.1002/clc.23370 (PMC7298972; doi:10.1002/clc.23370)
Supplement: Supplementary file 1 — Appendix S1: Supplementary Information [file CLC-43-546-s001.docx]

**Entry criteria**

Only patients with ECG-documented AF were included in the paroxysmal or (longstanding) persistent AF groups. Patients with documentation of atrial flutter were excluded from participation in the study. Criteria for hemodynamic instability included usage of inotropic agents or vasopressors and/or presence of a cardiac assist device. Emergency cardiac surgery was defined as cardiac surgery within 24 hours. Patients with end stage renal failure requiring dialysis were also excluded from participation in the study.

**Endpoints**

Primary endpoint of the study is development or recurrence of documented AF. In line with the latest ESC guidelines, AF is defined as an episode of at least 30 seconds with absolutely irregular RR intervals and no discernible, distinct P waves.

Secondary endpoints include:
- implantation of atrial pacemaker
- implantation of implantable cardioverter defibrillator
- withdrawal of informed consent
- lost to follow-up (unreachable via contact details of the home doctor or hospital)
- decease (confirmed by hospital, home doctor or Dutch BRP register)

**Study team members**

Translational Electrophysiology Research Unit of the Department of Cardiology at the Erasmus Medical Center, Rotterdam, The Netherlands:
- Natasja M.S. de Groot
- Paul Knops
- Maarten C. Roos-Serote
- Agnes Muskens-Heemskerk
- Annejet Heida
- Willemijn F.B. van der Does
- Lianne N. van Staveren
- Mathijs S. van Schie
- Corina Schram-Serban
- Rohit K. Kharbanda
- Charlotte A. Houck
- Ahmed A.Y. Ragab
- Danny Veen
- Lisette J.M.E. van der Does
- Christophe P. Teuwen
- Eva A.H. Lanters
- Gustaf D.S. Sitorius
- Roeliene Starreveld

Atrial Fibrillation Research Unit of the Department of Physiology at the Amsterdam UMC, Amsterdam, The Netherlands:
- Bianca J.J.M. Brundel
- Marit Wiersma
- Denise M.S. van Marion
- Kennedy S. Ramos
- Jin Li
